# A Passive Learning Sensor Architecture for Multimodal Image Labeling: An Application for Social Robots

**DOI:** 10.3390/s17020353

**Published:** 2017-02-11

**Authors:** Marco A. Gutiérrez, Luis J. Manso, Harit Pandya, Pedro Núñez

**Affiliations:** 1 Robotics and Artificial Vision Laboratory, University of Extremadura, 10003 Cáceres, Spain; lmanso@unex.es (L.J.M.); pnuntru@unex.es (P.N.); 2 Robotics Research Center, IIIT Hyderabad, 500032 Hyderabad, India; harit.pandya@research.iiit.ac.in

**Keywords:** robot sensors, ambient intelligence sensors, deep learning, object detection, object recognition, word semantics

## Abstract

Object detection and classification have countless applications in human–robot interacting systems. It is a necessary skill for autonomous robots that perform tasks in household scenarios. Despite the great advances in deep learning and computer vision, social robots performing non-trivial tasks usually spend most of their time finding and modeling objects. Working in real scenarios means dealing with constant environment changes and relatively low-quality sensor data due to the distance at which objects are often found. Ambient intelligence systems equipped with different sensors can also benefit from the ability to find objects, enabling them to inform humans about their location. For these applications to succeed, systems need to detect the objects that may potentially contain other objects, working with relatively low-resolution sensor data. A passive learning architecture for sensors has been designed in order to take advantage of multimodal information, obtained using an RGB-D camera and trained semantic language models. The main contribution of the architecture lies in the improvement of the performance of the sensor under conditions of low resolution and high light variations using a combination of image labeling and word semantics. The tests performed on each of the stages of the architecture compare this solution with current research labeling techniques for the application of an autonomous social robot working in an apartment. The results obtained demonstrate that the proposed sensor architecture outperforms state-of-the-art approaches.

## 1. Introduction

The possibilities and applications of autonomous social robots and ambient intelligence systems in our daily activities can be of utmost help. For the case of social robots, the research community has already studied how to perform several kinds of these tasks, such as cloth folding [1], floor vacuuming [2], cooking [3] or even more complex home assistant applications [4]. A basic skill on which these applications heavily rely is the need to identify and locate common household objects. Additionally, the problem of searching and finding objects extends to other automation applications requiring context awareness, e.g., Kidd et al. [5]. The complexity and variety in shape and location of these objects makes their detection one of the most complex skills to develop. Due to this complexity and despite the important advances in computer vision [6], most of the current solutions are still not robust enough to be applied in real household scenarios where clutter and changing environment conditions are common. Therefore, due to the importance of object detection for real household tasks, the development of methods to improve it has become a challenging and interesting research topic.

A property of indoor environments is that their items are usually structured hierarchically (e.g., an object lies on a table which is located in a certain room). Maintaining a representation of the environment and these hierarchical relationships can become extremely helpful when segmenting the environment. Additionally, objects in household environments are often found grouped by categories and placed relatively close according to their use, e.g., kitchen utensils are arranged together in the kitchen and toys are usually stored in a specific location in a certain room. These two properties can be exploited to successfully find and classify objects in indoor environments. Techniques such as cognitive subtraction [7] allow robots to use their world model in order to detect potentially unknown objects. The work presented here takes advantage of these properties and uses them to improve the detection of objects in household environments under real-world conditions.

**Figure 1:**
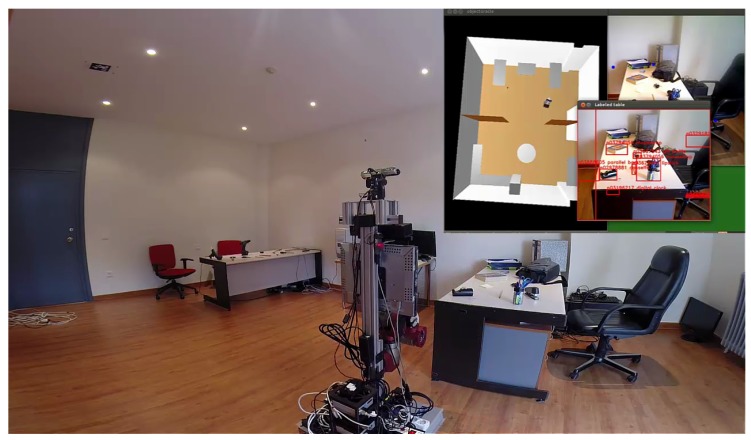
Shelly, a humanoid social robot, exploring an apartment and labeling objects on tables. The visualization of the labeling process is shown in the top right corner.

It has been demonstrated that long-term memory representations have a strong influence on human visual performance [8]. Therefore, taking advantage of previous experience through a long-term memory of a system is another key value that can be added to autonomous systems in order to improve the search of objects. The Passive Learning Sensor Architecture (PLSA) has been developed in order to exploit these advantages and to improve the performance when trying to find objects in large environments. It uses a world representation to filter the input data and focus the attention to those places where the objects of interest could be found. This information is obtained while the system is performing any task, even when it is apparently stopped (see the social robot use case in Figure 1). It is designed to be able to deal with low resolution images or light changes. Processing these images, the system learns the most probable object locations in order to help improve the time taken to find objects in later situations. The main contribution of the PLSA is that it improves the performance of social robots and autonomous systems in the task of finding objects in large environments through the combination of image labeling with word semantics. It uses multimodal information—language semantic information from trained models combined with visual input data—in order to estimate the most likely location for any given object. The system also exploits the existent semantic relationships among objects in the same object container to improve the final results. In the context of this paper, any object which can have objects inside or over it is considered an object container, i.e., a table, a shelf or a cupboard. However, in the use case presented here, a social robot searching for objects in an apartment, all the containers used are tables.

The pipeline of the architecture is composed of four main steps: a first cognitive attention stage to locate and segment object containers; a segmentation step to select smaller windows to label as objects; a Deep Neural Network (DNN) that is able to label the selected windows; and a final semantic matching step to improve and fine tune labeling results.

The use case of an implementation of PLSA in a social robot searching for objects in a 65 m${}^{2}$ apartment is presented for a better understanding. The robot learns from the images acquired while moving through the apartment during the execution of previous tasks. The PLSA takes advantage of its multimodality and combines these images with language semantic information to make early predictions on possible object locations. This way, the social robot is able to optimize the path to a successful search when searching for objects.

The remainder of this paper is organized as follows. After a brief summary about similar works in the literature in Section 2, Section 3 deeply explains the design of the PLSA. Section 4 describes the experiments that have been run to test the proposed sensor architecture. The conclusions and future lines of work are outlined in Section 5. For an easier understanding of the abbreviations used along the explanation of this work, a reference list is included at the end of the manuscript.

## 2. State-of-the-Art

The research community has demonstrated a high interest in the problem of active visual object search, however only a few works using semantic information have been published. Rangel et al. [9] presented a system to classify images using image descriptors generated from general purpose semantic annotations obtained through an external API. They propose using a semantic image descriptor of fixed dimension. Each entry of this descriptor corresponds to a label from a set of predefined labels and contains the probability of that label representing the image.

In [10], a dual 2D and 3D system to solve the search-and-find task is presented. The first 2D-based part of the system classifies wide scenes in order to decide whether to continue exploring in that direction. It uses texture information as the input for a DNN to automatically generate full descriptions from generic indoor images. These descriptions are syntactically and semantically processed in order to help a robot select which rooms to visit for a certain task. For the second part of the system, the 3D-based one, it takes advantage of the geometric information to look for specific objects within the scenes. Making use of 3D segmentation and classification pipelines, it is able to classify the different parts of the scene and locate specific objects. The approach presented in this paper is similar with this work in the sense that semantic relationships among words are used to predict the possible location of objects.

Semantic relationships are also exploited in [11] for object search in large environments. Uncertain environment spatial semantics are built and used in combination with imperfect semantic priors obtained from Internet databases in order to improve the efficiency of the search. This work differs from the one presented here in that it proposes reasoning about unexplored parts of the map, however it is similar to the PLSA in the sense that general semantic knowledge is used and applied in combination with extracted semantic cues to reason about locations of interest.

Regarding generic active object search, the work by Saidi et al. [12] should be pointed out. It models the search task as an optimization problem with the goal of maximizing the target detection probability while minimizing the distance and time to achieve the task. Visibility maps are computed based on a world model in order to search for a local maxima which is supposed to be the new sensor placement in the next step. To evaluate the interest of a given configuration multiple variables are used: the probability of detecting the object, the new volume that will be seen, and the cost in time and energy to reach that configuration. However, in contrast to the PLSA, no visual information is shared from one task to another or from previous experiences.

Another interesting solution exploiting object–object relations for active object search is presented in [13]. The work estimates object–room probabilistic relations that are extracted from a database and matched to defined ontological concepts in order to determine the next room to approach. These relations are used to compute the probability of finding the target object given a set of objects previously seen. This probability is then used to decide which object to approach next in the search process. This is similar to the PLSA in the sense that the relative location of objects to each other is exploited in order to optimize the search. However, the solution presented here differs in that it makes use of the semantic relationship among labels instead of probabilities as a measure of distance between objects.

Object search and localization of objects in indoor environments dealing with low resolution images is also addressed in [14]. The visual search mechanism is based on a combination of receptive field coocurrence histograms and the object recognition through SIFT features [15], while the view planning strategy takes into account the layout of the environment and the specific constraints of the object. They also improve concurrent multiple object search through an optimized use of their shared camera zooming. This work heavily relies on SIFT features, which are specific for object instances and cannot be directly used to detect general classes of objects. This could be extended with new state-of-the-art object recognition techniques (e.g., DNN-based solutions) to improve the accuracy and reduce the steps of the process.

## 3. Passive Learning Sensor Architecture

**Figure 2:**
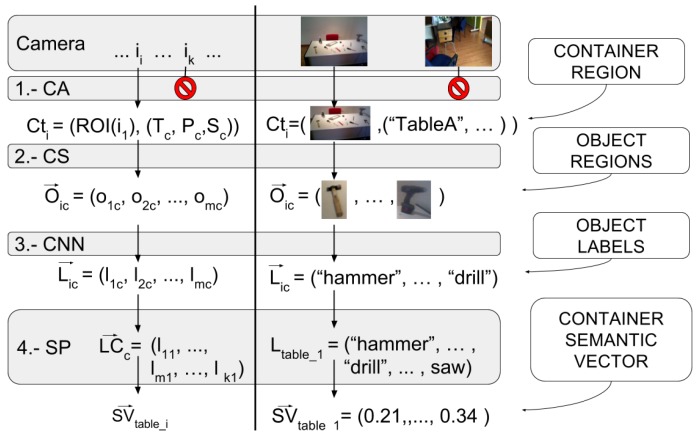
The four main steps of our passive learning architecture. The left-hand side of the vertical black line describes the output of each step in a mathematical notation, while the right-hand side shows it visually. Explanations on the outputs are given on the outer right descriptions. The forbidden sign means the image will be discarded.

The Passive Learning Sensor Architecture is designed to work in indoor environments, passively acquiring relatively low resolution images where the objects to detect are seen from a far distance. It detects possible object locations through multimodal information, combining semantic language data with image information. The architecture consists of a processing pipeline of four main steps, as illustrated in Figure 2. The first step, Cognitive Attention (CA), selects images that show a container with pontential objects in it. From these images, it extracts Regions of Interest (ROI), i.e., square parts of the image, where an object container is seen. The second one, called Cognitive Subtraction (CS), performs a segmentation of this ROI using the internal world model. This process extracts, within the regions selected during the first step, those regions potentially corresponding to known or unknown objects lying in the object containers. Afterwards, a Convolutional Neural Network (CNN) step sets labels according to the possible classes (i.e., object types) for the image regions obtained in the previous step. Finally, a Semantic Processing (SP) step uses a learned semantic model to improve the labels from the CNN and maximize the probability of finding the correct container for the object searched. After processing the images, it produces average semantic descriptors for each container found that will help in the later object search task. This section details the different stages of the pipeline of the sensor architecture. We use the case of a social robot looking for a mug to better explain the work flow of the architecture.

### 3.1. Cognitive Attention

This first stage of the architecture filters the images where an object container is seen, it detects regions of interest corresponding to object containers, and provides such regions along the information of the container in the image (identifier and geometrical properties) to the next stage of the pipeline. For each image, $I_{i}$ accepted; this first stage provides the next stage with a tuple $Ct_{i}$ containing the region of interest of the image that shows the container $ROI(I_{i})$, along with that container’s specific information such as its type (*T*), pose (*P*), and shape (*S*). See Equation (1).

(1) $$CA\left(i_{i}\right)=Ct_{i}=\left(ROI\left(I_{i}\right),\left(T,P,S\right)\right)$$

In order to decide if a container is seen (i.e., lies in the frustum of the camera), the architecture must have access to an estimation of the pose and shape of the containers. Given this information and the parameters of the camera, the first step is to check if any of the known object containers are seen in the current image and to estimate the area of the image occupied by it. This process of making the sensor focus its attention on the parts of the image in which containers can be seen is called Cognitive Attention (CA). Thanks to this step, the architecture only takes into account those pictures taken when a container lies within the frustum of the camera, and only processes the regions of the images showing those containers (in 1.-CA from Figure 2 you can see images going through and images not going through). This helps reduce the probability of false positives coming from parts of the environment that the architecture is not supposed to consider.

**Figure 3:**
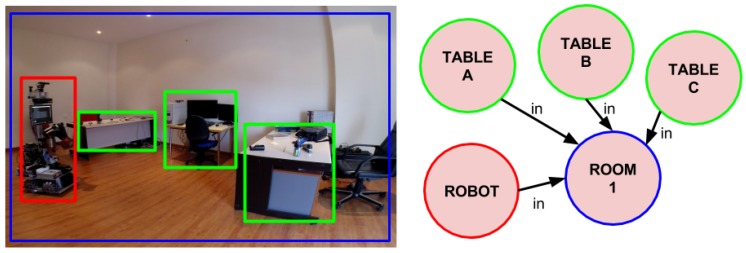
(**left**) A social robot uses the PLSA to search for objects in an apartment; (**right**) A schematic view of the AGM cognitive model, with the symbols of the robot, one room and three tables.

The cognitive model used in this implementation of the PLSA is Active Grammar-based Modeling (AGM) [16]. AGM cognitive models are multi-graphs where nodes and edges are typed and can be attributed with metric properties. The type of a node is used to denote the kind of entity it represents, whereas the type of an edge denotes the kind of relationship among the linked symbols. Metric properties can also be included in the nodes and in special edges designed to this end. The metric properties of nodes and edges allow to automatically generate a geometric model of the environment from the cognitive model. Additionally, these geometric models can be easily used to perform collision checks, to estimate relative poses and camera projections, and other operations. An AGM world model that contains the robot, rooms and tables is shown in Figure 3. However, other alternatives to this cognitive model can also be used (e.g., [17,18]) as long as they allow storing and accessing the necessary information, and support the computation of the contour of the containers in the images. Given the cognitive model, once an image is considered to have a container, the contour of that container is projected from 3D to the 2D space of the camera image. If at least 80% of the of the container lies within the image, then that region is selected and the next stage of the PLSA is triggered, otherwise the image is ignored.

Therefore, the CA stage selects the images from the camera that show containers and outputs the tuples corresponding to the detected areas of those containers along with the container information from the cognitive model. The output of the cognitive attention stage $CA(I_{i})$ is a vector of tuples $Ct_{i}$, where $Ct_{i}$ tuples are composed of the region of interest and the container information (i.e., its type, pose and size), as can be seen in Figure 2, 1.-CA step.

For the case of a social robot looking for a cup in the apartment where all containers are tables, this stage selects the images containing the tables as is moves around the apartment. It uses the information from its own world model to detect the location of the robot and the tables at any moment. From those images, this step selects the ROIs containing the tables and passes them along, with the shape and pose of the corresponding object container, to the next step.

### 3.2. Cognitive Subtraction

The second stage of the pipeline performs an additional segmentation step called Cognitive Subtraction (CS). It takes as input the tuples of the regions of interest obtained by the previous stage of the pipeline along with the container information, $Ct_{i}$, and generates a series of sub-regions of interest out of each container image, corresponding to possible objects $o_{jc}$. These sub-regions, which are expected to contain a single object each, are associated to its container and constitute the output of this stage, e.g., for one image, associated with a container *c*, it will produce a set of sub-images $\vec{O_{ic}}=\left(o_{1c},o_{2c},...,o_{mc}\right)$ (see 2.-CS in Figure 2):

(2) $$CS\left(Ct_{i}\right)=\vec{O_{ic}}=\left\{o_{1c},o_{2c},...,o_{mc}\right\}$$

Following the general idea suggested by Cotterill et al. [19], among many others, that rational behavior is “internally simulated interaction with the environment”, CS uses the cognitive understanding of the environment provided by the previous step in order to perform a proper segmentation of objects on the container. Thereafter, the CS algorithm uses the information about the object container and detects differences between the data acquired from the sensors and synthetic data obtained by *imagining* the output of the sensors, given the current world model.

The information obtained from the previous step contains the type, pose and size of the containers. The CS step triggers the specific algorithms to perform the subtraction of known elements in order to detect the new unknown ones. The following are the steps taken for the specific case of tables as containers in order to subtract the unknown data (unknown objects on the tables) from the real world data:

1. Random sample consensus [20] is used to estimate the plane of the table using the point cloud of the scene acquired with the RGB-D camera. The border of the table is estimated using a convex hull of the 3D points laying within the table plane. Using this border information, an imaginary prism is created on top of the table. All the 3D points inside this prism are extracted and considered to belong to the unknown objects lying on the table.
2. Different object point clouds are segmented using euclidean distance clustering [21]. A threshold distance to determine if points belong to the same object or to a new one is used (for the experiments conducted, 0.01 m).
3. Candidate object point clouds are transformed to image coordinates and the image region corresponding to the object candidate is segmented.

**Figure 4:**
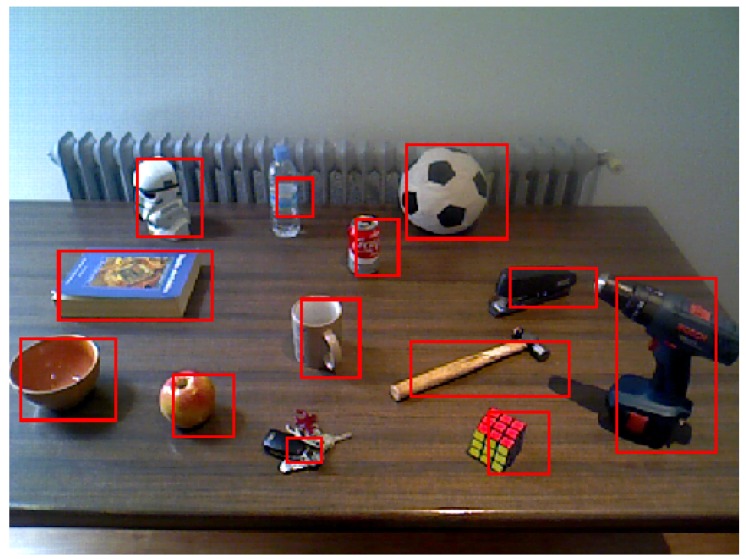
Example of segmentation by the cognitive subtraction algorithm. It must be taken into account that all the cognitive steps are made over the 3D data and projected back to the image.

Figure 4 illustrates a sample of the results obtained after these steps. The regions of interest, marked in the figure, correspond to parts of the environment that are unknown to the robot and are probably objects lying on the table. Therefore, they are fed to the next step for labeling purposes.

In the example of the social robot looking for a cup, the output of this step will be the segmentation of the objects lying on top of the table. Using the table selected along with the cognitive information from the previous step segmentation, the robot performs the specific steps to segment the objects on top of the table. The segmented parts, passed on to the next step, are the object candidates to be labeled.

### 3.3. CNN Classification Step

The third step of the PLSA classifies the image regions obtained from the previous step. It produces a label $l_{ic}$ for each of the object candidates regions $o_{ic}$ obtained in its input (see Equation (3) and 3.-CNN in Figure 2).

(3) $$CNN\left(\vec{O_{ic}}\right)=\vec{L_{ic}}=\left(l_{1c},l_{2c},...,l_{mc}\right)$$

Any algorithm able to perform this task can be used for this step. This processing of the object candidate regions is open to the usage of new algorithms that might, in the future, improve the current object classification state-of-the-art.

**Figure 5:**
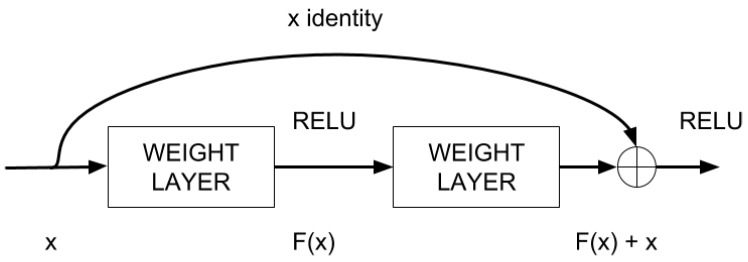
A building block of the residual learning process in our DNN.

The current implementation uses a very deep Convolutional Neural Network (CNN) based on deep residual learning [22]. The main characteristic of deep residual learning is the nature of their building blocks, illustrated in Figure 5. In the figure, F(x) represents the residual learning function, where *x* is the input of the layer *N* that gets added to the residual value at the layer N+1. This essentially drives the new layer to learn something different from what the input has already encoded. This is repeated for all the layers in the network.

The other advantage of this setup is that such connections help to handle the vanishing gradient problem in very deep networks, which slows down the training of front layers. This is defined formally in Equation (4); being *x* and *y* the input and output vectors respectively and having $F(x,W_{i})$ as the residual mapping to be learned. For the example in Figure 5 that has two layers, $F=W_{2}\sigma \left(W_{1}x\right)$ in which *σ* denotes the rectifier linear unit (RELU) [23].

(4) $$y=F(x,W_{i})+x$$

**Figure 6:**
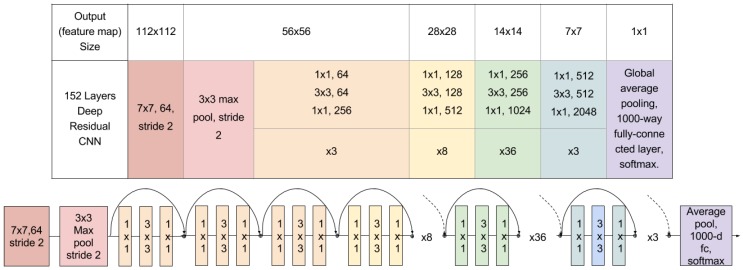
Architecture of the 152 layers CNN with residual learning.

Figure 6 shows the whole architecture of the 152 layers CNN with residual learning. At the top part of the figure, the network configuration is described while at the bottom it shows the graphical setup of the layers. Although the network used here is much deeper than others, it has a lower complexity (measured in FLOPs). While in a traditional approach, a layer has to generate a whole desired output, thanks to the design of the building blocks of the residual networks, their layers are only responsible for, in effect, fine tuning the output from a previous layer by just adding a learned residual F(x) to the input *x*. It uses 3-layer bottleneck blocks for which each residual function *F* uses a stack of three layers instead of two. The three layers are 1×1, 3×3, and 1×1 convolutions, where the 1×1 layers are responsible for reducing and then increasing (restoring) dimensions, leaving the 3×3 layer a bottleneck with smaller input/output dimensions.

Most of the time, training these networks is a heavy task in terms of time and hardware prerequisites. Therefore, the model of the CNN used in the experiments was trained using the generic ImageNet dataset. Also, this configuration proved to perform better than others tested here, as deeply explained in Section 4.

In the use case of the social robot, this step would only take the small images produced in the previous step, corresponding to objects on the table, and apply a label to each one of them. These labels, associated to their container, are then passed on to the next semantic processing step.

### 3.4. Semantic Processing

The Semantic Processing step (SP) takes all the labels produced by the previous step $L_{ic}$ for all the images and groups them according to their own containers *c*. Therefore, a vector of all labels from all images for each of the seen containers is processed separately $LC=L_{1c}+L_{2c}...L_{pc}=\left\{l_{c1},l_{c2},...,l_{cm},...,l_{ck}\right\}$. As a result, an average semantic vector $\vec{SV_{c}}$ is produced for each container *c* (see step 4.-SP in Figure 2).

Since the output of this step is a common vector for each container, all objects in the container will be considered when looking for a certain item. This process takes advantage of the fact that, in household environments, objects located next to each other are usually semantically related in some way or another. Sometimes, some mixing can be found but in general it can be said that they are usually grouped in spaces (toys, kitchen utensils, office supplies, etc.). As we use average semantic vectors to decide if the object is on a certain container, if an object is “out of place”, it might not be found on the first container selected. In the use case of the social robot, when looking for the cup, even if it is misplaced, the robot will go look for it in the kitchen, not around the tools or any other place, similarly to what a human might have done.

For this step, the skip-gram model [24] is used, also commonly known as *word2vec*. It is a “sallow” word embedding model which learns to map discrete words, represented by an id, into a low-dimensional continuous vector space using the distributional properties of the word observed along a raw text corpus (a large and structured set of texts). The word vectors learned can be used for different research purposes. One of the most common ones (the one used here) is the computation of the distance between word vector representations as a measure of word semantic similarity as, commonly, in the corpus provided in the training, similar words appear close to each other. The model used in this step for the current implementation of the PLSA is also a generic one. It has been trained on texts obtained from the Google News dataset (with more than 100 billion words).

This last step is intended to expand and improve the image labeling results by using the semantic relationships between the labels obtained on a certain container. Due to the low resolution of the images provided to the CNN step, the results obtained after it are still not robust enough for real applications. Additionally, the label used to identify the objects to search are often just a synonym or a similar word of the one the CNN is using, resulting in never finding the required object (e.g., the system provides the label *mug* to an object and the user is looking for a *cup*). This is also a case where semantics can help find a certain object (e.g., no *cup* has been detected but the detection of *coffee* guides the PLSA to the same container).

Vector representations help expand the semantics of the labels assigned to each container. Therefore, for every set of labels obtained for a certain container during the CNN step, $\vec{L_{ic}}$ an average Semantic Vector (SV) is computed $\vec{SV_{c}}$. This way, each container has a single semantic feature vector. These average vectors consider all labels in a certain location as a whole, minimizing the effect of false positives provided by the CNN step in the final object search.

Once the user of the system wants to find an object with label $l_{o}$, the semantic vector representation $SV_{l_{o}}$ of the label is computed using the learned skip-gram model. Afterwards, the Semantic Similarity SS to each container *c* is calculated as the cosine distance (dot product) of the representation of the label $SV_{l_{o}}$ and the semantic vector of the container $\vec{SV_{c}}$ (see Equation (5)). The higher the value obtained, the better result and the closer, semantically, the object searched is to an object container.

(5) $$\mathrm{SS}=\vec{SV_{l_{o}}}\cdot \frac{1}{n}\sum_{i}^{n}\vec{l_{ic}}=\vec{SV_{l_{o}}}\cdot \vec{SV_{c}}$$

**Figure 7:**
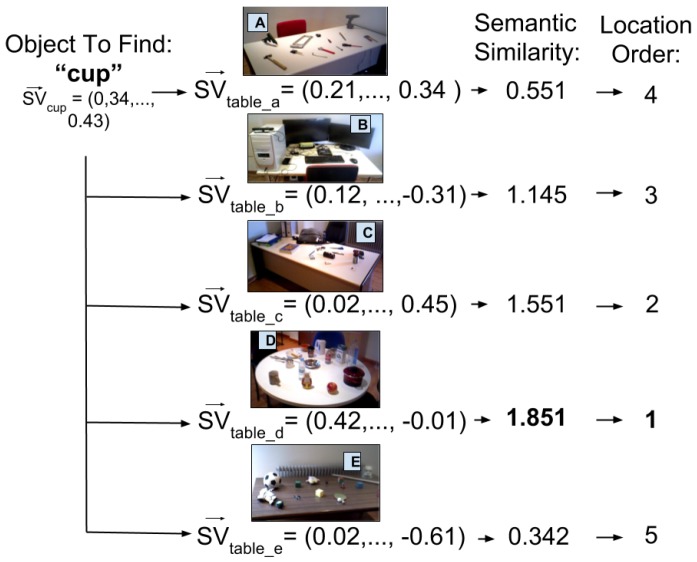
The process of finding an object location by obtaining the semantic similarity of a semantic vector with the known containers.

In the use case of the social robot wondering around the apartment, an average semantic vector representation would have been calculated for each of the tables. Afterwards, when looking for a *cup*, the semantic distance of the semantic vector representation of *cup* against all the vectors from the tables would be calculated. The higher the value of a table the more probable it is to find the object there. This is visually shown in Figure 7, as you can see the table selected to approach would be the one with the kitchen utensils.

## 4. Experiments

To demonstrate the effectiveness of all stages of the PLSA, each of them has been individually tested using a social robot. Additionally, an experiment has been conducted to study how the number of images used to inspect the containers affects the results.

**Figure 8:**
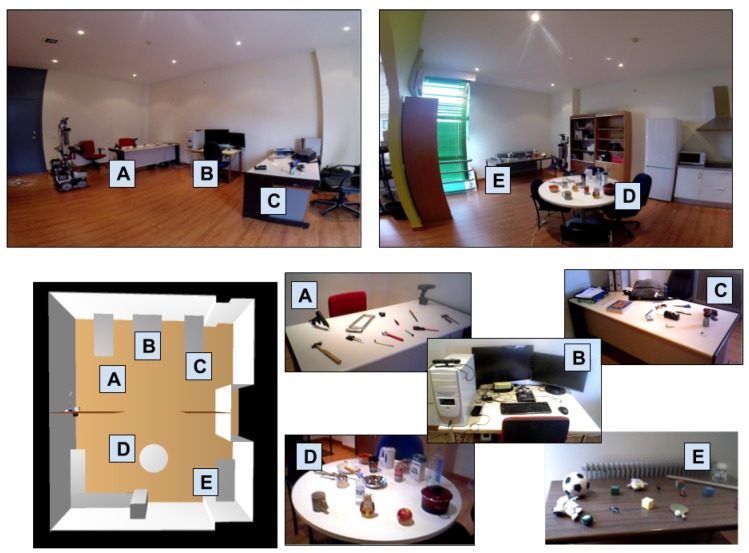
Setup of the experiment: On the top, the two room of the real setup; on the bottom-left, the visualization of the cognitive model of the setup; on the bottom-right, the tables and objects on them: table A contains tools, table B tech gadgets, table C office supplies, table D kitchen utensils and table E toys.

Figure 8 shows the environment in which the experiments took place. Five different object containers (tables in this case) are distributed among two rooms of an apartment, with five different types of objects on them. The disposal of the objects follows the principle that, in indoor environments, those with similar purposes are usually found in the same places. The tables in the apartment are configured for the experiments as follows (labeled as shown in Figure 8): table *A* contains hardware tools, table *B* has a computer and other tech gadgets, table *C* has office supplies, table *D* has kitchen utensils, table *E* contains different toys. It is worth noting that some of the objects are often labeled differently depending on the person asked (e.g., some people would label an object as a “toy” while others would call it a “plush”). Therefore, asking the robot about the location of an object using a particular label requires the system to be able to generalize.

The social robot is equipped with an RGB-D camera and operates in the apartment with an implementation of the PLSA. The experiments have an initial phase in which the robot wanders around the apartment, passively taking pictures of the tables, labels the objects lying on them and calculates the average vector representation of each container. Pictures are considered when a table is in the field of view of the camera, at less than 2 m and at a frame rate no higher than 1 Hz. These pictures are then used to train the average semantic vector representation of the tables. After the initial phase, the robot is asked to use the multimodal information (combination of images features and language semantics) to locate 20 objects among the ones on the tables. The results obtained are compared with the following combination of image segmentation and state-of-the-art CNN image recognition systems:

- GoogleNet [25] is a 22 layers deep network (27 if pooling is taken into account) that makes use of “inception modules” which basically act as multiple convolution filter inputs, that are processed on the same source, while pooling at the same time. Another training of this network but without relighting data-augmentation was also tested (GoogleNet2).
- AlexNet, by Krizhevsky et al. [26], consists of eight layers, of which five are convolutional layers, with some of them being followed by maxpooling layers. The other three layers are fully-connected layers with a final 1000-way softmax.
- Very Deep Convolutional Networks by Simonyan et al., presented in [27] (VGG16) consist of a series of thirteen convolutional layers (also with maxpool in between), followed by three fully connected layers.
- Regions with Convolutional Neural Network (R-CNN) [28] performs localization and classification of the objects in the image. It generates category-independent region proposals, then a convolutional network extracts a fixed-length feature vector from each region and finally the third module, which is a set of class-specific linear SVMs, scores each feature vector. Since it performs localization by itself, no previous segmentation step is added to this network.

These networks competed in the ImageNet Large-Scale Visual Recognition Challenge (ILSVRC) [29] and the PASCAL VOC [30] competitions and obtained top positions in their rankings. In the experiments presented in this section, they were tested with both, generic and specific training data.

The following experiments are as follows. Section 4.1 tests how the number of images processed per container affects the whole system. This is done to look for, if it exists, a maximum number of images after the PLSA should stop processing for a certain container. For this, we watch the results of the whole PLSA as we add images to a certain container. Afterwards, the Cognitive Attention step is tested by comparing the results of the PLSA with this stage and without it, in Section 4.2. In Section 4.3, the output after the the first three layers and the whole architecture is compared with combinations of previously mentioned state-of-the-art algorithms. This proves not only that the results of the three first layers improve state-of-the-art algorithms but also that they improve when adding the final Semantic Processing step. Finally, Section 4.4 shows how specific retrains of these networks do not actually improve the results of the PLSA, which justifies the use of generic training data sets for the architecture models.

### 4.1. Tests on Image Buffering

**Figure 9:**
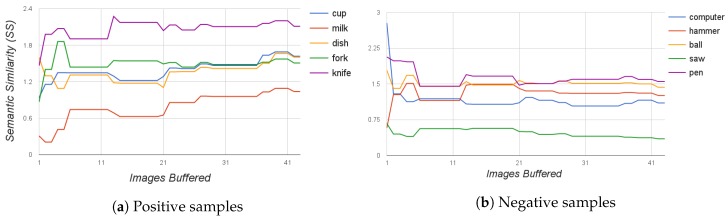
These graphs show how the semantic similarity between objects and the SV of table A evolves as more images of table A are added to the buffer of the PLSA. The higher the Semantic Similarity value is, the higher the likelihood of the presence of the object. (**a**) objects queried are present in the table; (**b**) objects queried are not present in the table.

The purpose of this test is to study the evolution of the Semantic Similarity SS of an object to the containers as the size of the PLSA image buffer is increased. The number of labels produced and taken into the semantic step increases as more images are added to be processed. This can make the final results vary. To test this, results were obtained starting from a buffer size of one image, and the size was increased one by one up to a buffer size of 43 images. Figure 9 shows how the semantic similarity of the labels of objects with table A evolves as images of such a container are added to the buffer. In Figure 9a, only objects present on the table are tested against the labels from table A. As a general rule, it can be said that the similarity with labels of present objects keeps increasing or stabilizes as images are added. The contrary occurs in Figure 9b, where objects not present on the table are queried for table A. In this last case, similarity decreases as images are added. Therefore, it can be concluded that adding images to the buffer either improves or it does not negatively affect the results of the architecture. Little improvement is appreciated for a buffer size higher than 20 images. Because of these results, during the rest of the experiments, the buffer size was set to store the information of the last 30 images for each object container.

### 4.2. Cognitive Attention Tests

**Figure 10:**
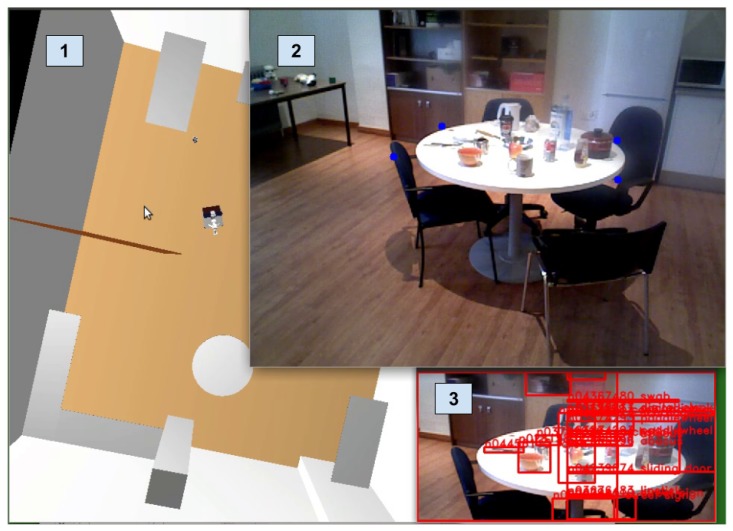
Cognitive attention in practice: **1**. Shows the visualization of the cognitive model; **2**. Is the real Primesense sensor camera with augmented reality, projecting the cognitive location of the table marked with four blue dots; **3**. Shows the specific part of the image to process along with a Top-Hat based segmentation and a GoogLeNet classification.

To test the Cognitive Attention stage, this experiment compares the results obtained with and without this stage. The setup of the experiment is depicted in Figure 10. Table 1 shows the results obtained. Using the Cognitive Attention step, only the table region of the image is considered, while without it, the full image is used. Results prove the effectiveness of this step as the PLSA obtains a higher success rate when using CA.

Additionally, to prove that CA is not only beneficial to the PLSA, this step was also tested with another segmentation and labeling technique: the Top-Hat (TH) [31] combined with three of the main DNN presented at the beginning of Section 4. As can be appreciated in the table, the results are equal or better when focusing on the selected table region of the image.

### 4.3. Tests with Networks with Generic ImageNet Training

The full PLSA was compared with a combination of state-of-the-art object detection and recognition systems. For this, three main segmentation algorithms were used:

- Top-Hat [31]: A morphology transformation based algorithm commonly used for segmentation purposes.
- Multiscale Combinatorial Grouping [32]: An algorithm for bottom-up hierarchical image segmentation.
- The Cognitive Subtraction: Algorithm explained in Section 3 and stage two of the PLSA.

These segmentation algorithms were combined with the CNN architectures with best results in the top challenges in computer vision, addressed in Section 4. To test the system, it was queried with 20 objects and asked to locate them among the five object containers considered for the experiment (tables *A*, *B*, *C*, *D* and *E*). If the first choice of the algorithm was the correct table, it was counted as a success, otherwise it was considered a failure.

The first three steps of the PLSA are compared with a mix of the segmentation algorithms combined with the DNNs explained configurations with generic trainings. Table 2 shows the normalized success rate of all of them. For results on the second column (Direct Match), a direct naive match, in which labels of objects to find are directly matched with the labels detected in the obtained images, is used. The third column shows the results when adding the semantic processing step. The results prove how the PLSA outperforms all of the other solutions tested and, at the same time, how a semantic processing step brings improvements not only to PLSA but also, in most of the cases, to the labels resulting from the rest of the CNNs. All the DNN algorithms used for the results in Table 2 use models trained with the generic ImageNet database.

### 4.4. Tests with Networks with Fine-Tuned Training Datasets

These experiments perform the same tests as the previous one, but using fine-tuned data sets. Instead of using the models trained with the generic ImageNet database, a specific fine-tuned retrain on a reduced set of classes was used. For these tests, the following changes were made to the models of the DNNs:

- GoogLeNet2_ft: this model is the ImageNet trained GoogLeNet2 model with a retrain on the last full-connected layer for 138 classes.
- VGG16_fc1: is VGG16 fine-tuned on 1000 categories by simply training on new images.
- VGG16_fc2: uses the VGG16 model but retraining the last fully-connected layers on 136 categories.
- VGG16_fc3: is VGG16 fine-tuned on 44 categories by changing the last fully-connected layer.
- R-CNN_m: is using the pretrained R-CNN with bounding boxes (region proposals) given by MCG instead of Selective Search.

As well as in Table 2, the first three steps of the PLSA are compared to different combinations of segmentation algorithms and DNNs, and then the Semantic Processing step is added to all the combinations to test its effectiveness. The resulting success rates are shown in Table 3. They prove how the PLSA (even with a generic training) still outperforms the success rates of fine-tuned networks. However, it is worth noting that the semantic processing step does not perform as good as on the previous tests with these training configurations.

In general, the success rates of the direct match approach (without the Semantic Processing step) show better results for these retrained networks than for the generic ones. However, when adding the semantic processing step, these success rates do not seem to improve, they even worsen in some cases. In order to further investigate why this step does not improve the networks with fine-tuned models, some extra experiments were performed. The average semantic similarity of the labels applied to each container were computed. Average values of the results of models trained with the generic database and the retrained models were compared. Results of these computed average semantic similarity are shown in Table 4. For each table, an average semantic distance among all the vector representations of the labels found for that table was computed. Since the semantic similarity is an inverse distance measure, a higher value means the label representations of a table are “semantically closer” in the search space. For all the cases, the average similarity among all the labels applied to tables in the generic trained networks is higher than the similarity for the same networks when retrained with a lower amount of classes. These results mean that labels applied to containers by the generic training are closer to each other in the search space than the ones from the fine-tuned trainings, which means they are also semantically closer.

**Figure 11:**
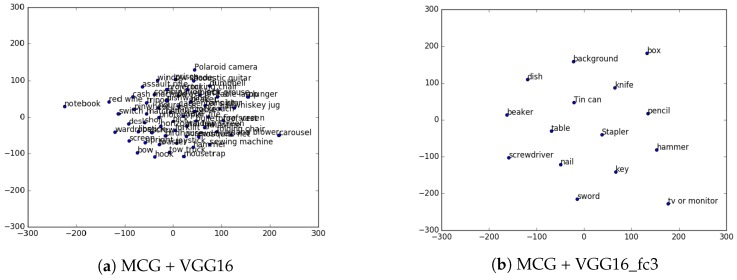
Visualization, using t-SNE for dimensionality reduction, of labels applied to table A.

For a better visualization of this issue, t-SNE [33] was used. It is a dimensionality reduction technique well suited for the visualization of high-dimensional datasets. Using this technique, Figure 11 shows a plot of the vector representations of labels obtained for table A, when using a generic training (Figure 11a) and when using a fine-tuned one (Figure 11b). Since generic trainings have more classes, sometimes they generate *light fails*. A *light fail* is a name used to denote failure made with a word that is not very far in meaning to the correct one (e.g., mug instead of cup). This light fails to help maintain the $\vec{SV}$ of the container around the similar space, almost as if it were a correct answer, therefore not considerably affecting the final result. However, when using the models retrained with a reduced set of classes, and probably influenced by the lack of vocabulary available in their results, the fails are usually not so *light*. This means that a fail, more often than with the big ImageNet generic trained models, will be a totally different word (e.g., *screwdriver* instead of *cup*). This makes the location in the search space of the $\vec{SV}$ of a container very affected by the fails of these models, which makes it have a lower semantic similarity (a higher distance to the words in the search space) to the actual correct labels of the objects it contains, lowering its general success rate. Therefore, it can be concluded that generic training datasets with a larger number of classes are more likely to benefit from the semantic processing step of the PLSA.

## 5. Conclusions and Future Work

The PLSA was introduced and deeply described. The experimental results obtained from its use in a household social robot were also presented. It was demonstrated that it outperforms state-of-the-art algorithms both with generic and fine-tuned training datasets. The different steps of the architecture were deeply tested, showing how they contribute to the whole architecture and to its final performance. It is believed that this solution constitutes a firm candidate to be used as a first step to guessing object locations either in social robotics tasks or any other solution that involves search functions in broad scenarios.

Further work improving the different steps in the architecture can be made. Specially, it would be interesting to explore new alternatives for the semantic processing step. Other word semantic relationships can be tested in an effort to improve this process. It can also help discover what specific training setups can actually benefit from it. Different strategies for selecting specific labels that might become representative of certain locations might help improve results.

The current output of the system only handles one query at a time and this may also be extended. Taking into account multiple object searches at the same time might help reduce the time of high level tasks with a strong dependence on the find-and-search process, e.g., having a robot clean an apartment or cook something in the kitchen. For these tasks with multiple objects, instead of one container, a best path to find several objects should be given as an output. Therefore, the distance between containers should also be taken into account.

This architecture is meant as a first guess on where to look for an object. Further work might be required to strengthen final results and have actuators that actually verify the existence of the object or even robots that fetch and bring the item to a specific location. Current implementation of the PLSA provides a primary guess in the search task; in order to fully integrate the architecture into higher level plans, more search and verification steps will be needed to assure a final successful execution of the plan. For example, when two objects with the same label are found, an extra step could be added in this verification step to decide which one to choose.

## Figures and Tables

**Table 1:** Success rate of the PLSA and other algorithms’ configurations when using full images of the ROI obtained from the Cognitive Attention step.

Method	Full Image	Cognitive Attention
PLSA (no semantics)	0.4	0.65
TH + GoogLeNet	0.35	0.35
TH + GoogLeNet2	0.35	0.35
TH + AlexNet	0.2	0.35
TH + VGG16	0.55	0.6

**Table 2:** Success rate of the object search test when using the first three steps of the PLSA against top CNN algorithms and results when adding the SP step.

Method	Direct Match	Semantic Processing
PLSA	0.65	0.75
TH + GoogleNet	0.35	0.45
TH + GoogleNet2	0.35	0.45
TH + AlexNet	0.35	0.1
TH + VGG16	0.6	0.6
MCG + GoogleNet	0.5	0.5
MCG + AlexNet	0.15	0.25
MCG + ResNet	0.55	0.55
MCG + VGG16	0.55	0.45
CS + GoogleNet	0.45	0.5
CS + GoogleNet2	0.6	0.65
CS + AlexNet	0.2	0.25
CS + VGG16	0.45	0.45
R-CNN	0.4	0.0

**Table 3:** Average success rate of the object search test with the PLSA and fine-tuned networks. The second column shows the results of not including Semantic Processing and the third one shows the results when including this step.

Method	Direct Match	Semantic Processing
PLSA	0.65	0.75
TH + VGG16_fc1	0.5	0.4
TH + VGG16_fc2	0.65	0.45
TH + VGG16_fc3	0.4	0.4
TH + GoogleNet2_ft	0.35	0.6
MCG + VGG16_fc1	0.5	0.4
MCG + VGG16_fc2	0.55	0.55
MCG + VGG16_fc3	0.55	0.4
MCG + GoogleNet2_ft	0.35	0.4
CS + VGG16_fc1	0.65	0.6
CS + VGG16_fc2	0.6	0.5
CS + VGG16_fc3	0.5	0.5
CS + GoogleNet2_ft	0.45	0.4
R-CNN_m	0.35	0.2

**Table 4:** Average word2vec semantic similarity between labels detected per table. Rows in bold correspond to the original models without fine tuning.

Method	Table A	Table B	Table C	Table D	Table E
**MCG + VGG16**	**0.6014**	**0.3906**	**0.37714**	**0.33766**	**0.4097**
MCG + VGG16_fc1	0.19741	0.23967	0.19976	0.21568	0.28264
MCG + VGG16_fc2	0.18415	0.22191	0.18938	0.17405	0.24179
MCG + VGG16_fc3	0.2178	0.19943	0.18505	0.20464	0.23815
**MCG + GoogLeNet2**	**0.28346**	**0.2965**	**0.24557**	**0.2136**	**0.36935**
MCG + GoogLeNet2_ft	0.18915	0.19017	0.18314	0.18309	0.32282

## Supplementary Materials

A video of the robot setup and run can be found at: https://youtu.be/7dWIc5zc8vo. All the code involved in the experiments are part of the RoboComp framework which is freely available at: https://github.com/robocomp.

## Abbreviations

The following abbreviations are used in this manuscript:

AGM	Active Grammar Model
API	Application Programming Interface
CA	Cognitive Attention
CNN	Convolutional Neural Network
CS	Cognitive Subtraction
DNN	Deep Neural Network
FLOP	Floating Point Operations
GPU	Graphic Process Unit
ILSVRC	ImageNet Large-Scale Visual Recognition Challenge
MCG	Multiscale Combinatorial Grouping
PLSA	Passive Learning Sensor Architecture
R-CNN	Regions with Convolutional Neural Networks
RELU	Rectifier Linear Unit
RGB-D	Red Green Blue Depth
ROI	Region Of Interest
SIFT	Scale Invariant Feature Transform
SP	Semantic Processing step
SV	Semantic Vector
SVM	Support Vector Machine
TH	Top-Hat
t-SNE	t-distributed Stochastic Neighbor Embedding
VGG	Visual Geometry Group
VOC	Visual Object Challenge
